# Non-Woven Fibrous Polylactic Acid/Hydroxyapatite Nanocomposites Obtained via Solution Blow Spinning: Morphology, Thermal and Mechanical Behavior

**DOI:** 10.3390/nano14020196

**Published:** 2024-01-15

**Authors:** Javier González-Benito, Stephania Zuñiga-Prado, Julian Najera, Dania Olmos

**Affiliations:** 1 Department of Materials Science and Engineering and Chemical Engineering, Instituto de Química y Materiales Álvaro Alonso Barba (IQMAA), Universidad Carlos III de Madrid, Avda. Universidad 30, 28911 Leganés, Madrid, Spain; javid@ing.uc3m.es (J.G.-B.); stzuniga@ing.uc3m.es (S.Z.-P.); 2 Instituto de Química y Materiales Álvaro Alonso Barba (IQMAA), Universidad Carlos III de Madrid, Avda. Universidad 30, 28911 Leganés, Madrid, Spain; 3 Department of Aerospace & Mechanical Engineering, University of Notre Dame, Notre Dame, IN 46556, USA; jnajera2@nd.edu

**Keywords:** polylactic acid, hydroxyapatite, nanoparticles, polymer nanocomposites, solution blow spinning

## Abstract

Polylactic acid (PLA) is widely used in tissue engineering and other biomedical applications. PLA can be modified with appropriate biocompatible ceramic materials since this would allow tailoring the mechanical properties of the tissues to be engineered. In this study, PLA-based non-woven fibrillar nanocomposites containing nanoparticles of hydroxyapatite (HA), a bioceramic commonly used in bone tissue engineering, were prepared via solution blow spinning (SBS). The compositions of the final materials were selected to study the influence of HA concentration on the structure, morphology, and thermal and mechanical properties. The resulting materials were highly porous and mainly constituted fibers. FTIR analysis did not reveal any specific interactions. The diameters of the fibers varied very little with the composition. For example, slightly thinner fibers were obtained for pure PLA and PLA + 10% HA, with fiber diameters of less than 400 nm, while the thicker fibers were found for PLA + 1% HA, with average diameters of 427 ± 170 nm. The crystallinity and stiffness of the PLA/HA composite increased with the HA content. Further, composites containing PLA fibers with slightly larger diameters were more ductile. Thus, with an appropriate balance between factors, such as the diameter of the solution-blow-spun PLA fibers, HA particle content, and degree of crystallinity, PLA/HA composites may be effectively used in tissue engineering applications.

## 1. Introduction

The preparation of materials composed of small-diameter fibers is recently attracting significant attention due to their potential as scaffolds for tissue engineering [1,2,3,4], membranes [5], filters [6], sensors [7], and drug-release systems [8,9], among others. The properties of these materials can be tuned via variables such as the nature of the components, composition (blends or composite materials), structure, and morphology. While the effect of component choice on the final material properties is relatively well understood, those of the composition, structure, and morphology are less well-known and may be more complex. Therefore, it is essential to direct more significant efforts towards investigating these aspects. 

Notably, within the field of fiber-based materials, special attention is dedicated to those designed for scaffold construction in tissue engineering and regeneration [10,11]. The increasing need for organs has necessitated the reconstruction of organs and tissues, resulting in the foundation of a discipline known today as ‘tissue engineering’ (TI) [10]. According to Langer and Vacanti [11] ‘tissue engineering’ is an interdisciplinary field that applies the principles of engineering and life sciences toward the development of biological substitutes to restore, maintain or improve the function of a biological tissue or an entire organ”. The objective is not to transplant organs but to find an appropriate method to recover the functionality of a damaged organ or tissue, for instance, integrating a laboratory-manufactured tissue (using scaffolds) into the body or active materials, which favors tissue regeneration.

Most materials used to engineer osseous tissue are polymers, bioactive ceramic materials, and/or composite materials; the polymers can be natural or synthetic [12,13,14,15,16]. Natural polymers such as fibrin, chitosan, or collagen are interesting candidates because they exhibit good compatibilities and osteoconductivities and low tendencies to generate immune responses (immunogenicity) [17,18]. In contrast, synthetic polymers enable the generation of matrices with more homogeneous and tuned properties. Within the framework of biodegradable and bioresorbable polymers, systems based on polyhydroxyalkanoates (PHAs) have been widely used in tissue engineering, owing in part to their approval by the European and American drug agencies [19,20]. Examples of PHAs are polylactic acid (PLA), polyglycolic acid (PGA), and polyhydroxybutyrate (PHB).

PLA has a longstanding history in biomedical applications, and its use has been approved by the US Food and Drug Administration (FDA) since 1970 [19]. Since its approval by the FDA for use in the human body [13], PLA has been mainly used in wound management, orthopedic and fixation devices, drug delivery, and tissue engineering. In particular, the polymer has been used to aid dental extraction wound healing, improve surgical sutures, and replace traditional metal implants to promote a gradual recovery. The polymer has also been used as a drug host to allow their continuous and controlled release via hydrolytic ester cleavage and erosion, swelling, and diffusion, and serves as a scaffold for tissue regeneration [17]. However, many of these applications do not employ PLA alone; instead, composites and blends of PLA with other materials as copolymers are used to improve the final performance of the materials [12].

One of the most effective strategies for creating porous polymeric matrices with the desired mechanical and physicochemical properties relies on the use of composite materials [21,22]. The incorporation of ceramic particles can help to tailor the mechanical properties for them to be like those of the tissue to be regenerated. For example, mesoporous silica can be used for bone tissue engineering [23]. Hydroxyapatite (HA) and its derivatives and combinations are some of the most common bioceramic materials used in bone tissue engineering (BTE) [24,25,26,27]. This is because their surface properties favor adhesion, differentiation, and cell growth, and they can bind and concentrate bone morphogenetic proteins in vivo. Thus, over the past three decades, HA, similar to the mineral component of natural bone, has been widely studied and is now used in BTE [22].

Ensuring the uniform dispersion of nanofillers within a polymer matrix is crucial for controlling material properties. However, achieving complete uniformity can be challenging. To date, various methods have been proposed to prepare polymer nanocomposite materials, such as solvent casting [28], modification of the polymer matrix [17], surface modification of the nanoparticles (NPs) [18], and even more recently, additive manufacturing [16]. In this study, to satisfy this purpose, solution blow spinning (SBS) is proposed as an alternative method to electrospinning (ES) or additive manufacturing (AM) for the preparation of fibrillar materials in biomedical applications, given that there is no need to use an electric voltage or high processing temperatures. This versatile method employs a concentric nozzle, wherein the polymer solution is injected through the inner nozzle, while a high-pressure air flows through the outer nozzle to exert pressure on the polymer solution at the nozzle exit. Once the first droplet of polymer solution is formed at the tip of the inner nozzle, it is stretched by pressurized air, which helps solvent evaporation and may induce fiber formation if adequate processing conditions are used. Finally, the material is deposited on a collector located at a certain distance (working distance) from the nozzle [29,30,31,32,33,34,35,36]. Despite challenges and potential limitations, such as fiber alignment or orientation [36], the simplicity, fast processing, and versatility of SBS make it an attractive choice for nanofiber scaffold fabrication in various applications. Although further research is still needed to explore the influence of SBS parameters on the final material structure, these advantages position SBS as a promising method for achieving controlled material properties in nanocomposites. A thorough understanding of the materials’ morphological, surface, thermal, and mechanical properties is necessary for their effective applications and potential uses. 

A comprehensive understanding of the materials’ morphological, surface, thermal, and mechanical properties is essential for effective applications and potential uses. In light of these considerations, in this study, PLA-based polymer nanocomposites filled with HA NPs were prepared via SBS. Dispersions of NPs in dichloromethane polymer solution were prepared to be blow spun for manufacturing polymer nanocomposites with varying concentrations of HA (0, 1, 2, 5, and 10 wt.%). The structure and morphology of the materials, as well as thermal and mechanical performance, were studied as a function of their composition and processing conditions, looking for correlations that allow tailoring made materials for different applications. Specifically, the outcomes of this research will facilitate a comprehensive exploration of the relationships between material composition (HA content), fiber diameter, crystallinity, and stiffness within materials produced via SBS. These insights will provide new possibilities for developing materials optimized for tissue engineering purposes. 

## 2. Materials and Methods

### 2.1. Materials

PLA manufactured by Nature Works LLC (Ref. code: PLA Polymer 7032D; glass transition temperature, *T*_g_ = 55–60 °C; melting temperature, *T*_m_ = 160 °C; and processing temperature = 200–220 °C) was acquired from Resinex Spain, SL. Hydroxyapatite, HA (particle size (diameter) ≤ 200 nm) was acquired from Sigma Aldrich (PCode: 1002598785) and used as a filler to prepare PLA/HA nanocomposites. The structure and morphology of the hydroxyapatite were studied in a previous work [37] and are in good agreement with the information provided by the supplier. Dichloromethane (purity = 99.9%, Sigma Aldrich, St. Luis, MO, USA) was used as the solvent to prepare the polymer solutions and particle suspensions.

### 2.2. Sample Preparation

Suspensions of HA NPs in PLA solutions (25 mL) were prepared by suspending various concentrations of HA NPs (0, 1, 2, 5, and 10% wt) in a 10% *w*/*v* PLA solution in dichloromethane. To prepare the nanocomposite materials, we use the following protocol: weigh 2.5 g of polylactic acid (PLA) in a vial, dissolve it in 12.5 mL of dichloromethane (CH_2_Cl_2_), and seal it. After 24 h, prepare a suspension with the necessary amount of hydroxyapatite (HA) nanoparticles in 10 mL of CH_2_Cl_2_. Next, sonicate the HA suspension in an ultrasound bath at room temperature for 30 min to achieve nanoparticle dispersion and create a homogeneous suspension. Once sonication is complete, add the HA suspension to the prepared PLA solution. Utilize 2.5 mL of CH_2_Cl_2_ to clean the vial containing the HA suspensions and clean any possible remaining HA nanoparticles. Incorporate it into the prepared PLA solution, bringing the total volume to 25 mL to attain the desired concentrations of PLA (10% *w*/*v*) and HA nanoparticles. To maintain the homogeneity of the PLA/HA suspensions, continuous stirring was done until the initiation of the solution blow spinning (SBS) process.

**Scheme 1:**
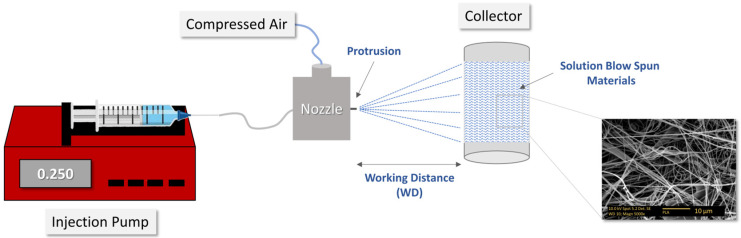
Schematic representation of the solution blow spinning device and conditions used to prepare the PLA/HA materials.

Polymer film samples were prepared via SBS and collected on a rotating cylindrical surface covered by aluminum foil. Using a ten-milliliter syringe, 25 mL of each suspension was injected. Notably, the remaining portion of the solution was maintained under continuous agitation until its subsequent use in the process. A 0.5-millimeter-diameter needle was used to feed a concentric nozzle to carry out the SBS process. The nozzle of this equipment comprises two tubes: a perforated aluminum tube (1 mm) for gas flow and a glass tube (0.5-mm inner diameter with a wall thickness of 0.2 mm) for polymer solution. A lateral hole (4.0 mm) is used for introducing gas. The inner tube protrudes 2 mm from the outer tube. This protrusion is one of the parameters that can affect fiber formation and material morphology. Scheme 1 illustrates the solution blow spinning setup for preparing PLA/HA nanocomposite materials, and Table 1 presents the corresponding SBS processing conditions.

### 2.3. Characterization Techniques

Morphological characterization was performed via scanning electron microscopy (SEM) using a Philips XL30 SEM microscope (Oxford Instruments Pte Ltd., Techlink, Singapore). The samples were gold coated by sputtering using a Leica EM ACE200 low-vacuum coater (Fraction Technologies, Changi Business Park, Singapore). Elemental composition analysis was conducted using Energy-Dispersive X-ray Spectroscopy (EDS) (Oxford Instruments Pte Ltd., Techlink, Singapore) to identify the HA-rich domains and their distribution in the polymer matrix. Infrared spectroscopy was used as a complementary technique for the initial characterization of the materials. Measurements were conducted utilizing the ThermoFisher Nicolet iS 5 spectrometer (Thermo Fisher Scientific, Waltham, MA, USA) with an ATR device equipped with a diamond window known as GladiATR from PIKE Technologies (Madison, WI, USA). Spectra of the samples were collected from 400 to 4000 cm^−1^, with 32 scans and a resolution of 4 cm^−1^.

Thermal characterization of the materials was performed using differential scanning calorimetry (DSC) and thermogravimetric analysis. DSC experiments were conducted in a Mettler Toledo DSC822e calorimeter (Mettler-Toledo GmbH, Greifensee, Switzerland). The thermal program carried out consisted of (i) a first heating scan from 35 to 200 °C at 10 °C·min^−1^, (ii) 5 min at 200 °C, (iii) a cooling scan from 200 to 35 °C at 10 °C·min^−1^, (iv) 5 min at 35 °C, and (v) a second heating scan from 35 to 200 °C at 10 °C·min^−1^. The thermal transitions of the polymer (glass transition, melting, and crystallization) were analyzed during the heating and cooling scans. The degree of crystallinity was calculated from the heating scans using the following equation:

(1) $$\chi =\frac{\Delta H_{m}-\Delta H_{cc}}{\Delta H_{m}\left(100\%\right)}\times 100$$

where Δ*H*_m_ and Δ*H*_cc_ represent the melting and cold-crystallization enthalpies, respectively, and ΔH_m_ (100%) is the melting enthalpy of 100% crystalline PLA for which the reported value of 93.6 J·g^−1^ [38,39] was used.

**Figure 1:**
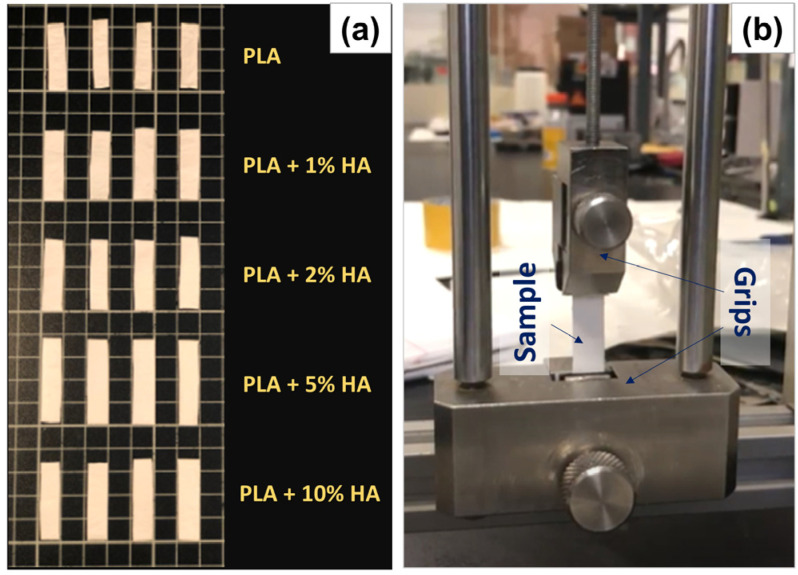
(**a**) Rectangular specimens prepared for mechanical tests; (**b**) sample placed between clamps of the testing machine.

Mechanical characterization of PLA and PLA/HA nanocomposite materials was performed through tensile tests using a Microtest universal testing machine employing SCM3000 software. The polymer samples were prepared by cutting four rectangular specimens of dimensions 30–40 mm × 7–9 mm from each type of material (Figure 1). Average values of the length, width, and thickness, respectively, were obtained for each sample. For each specimen, the thickness was obtained from the average of three measurements taken at three different points on the specimen using a caliper. Also, the specimens were weighted to calculate their apparent densities, which were compared with the bulk densities of the materials. For each sample, the densities of the bulk materials were calculated using the rule of mixtures [40,41]. 

Before positioning the specimens on the testing machine, rubber pieces were attached to the surface of the clamps to prevent the samples from slipping during the tensile test. Once properly loaded onto the clamps, a pre-loading force of approximately 0.05 N was applied to the samples, and the initial distance between the clamps was measured using a digital caliper. The specimens were then mechanically tested by applying a traction velocity of 1.0 mm·min^−1^.

## 3. Results and Discussion

### 3.1. Structural and Morphological Characterization

**Figure 2:**
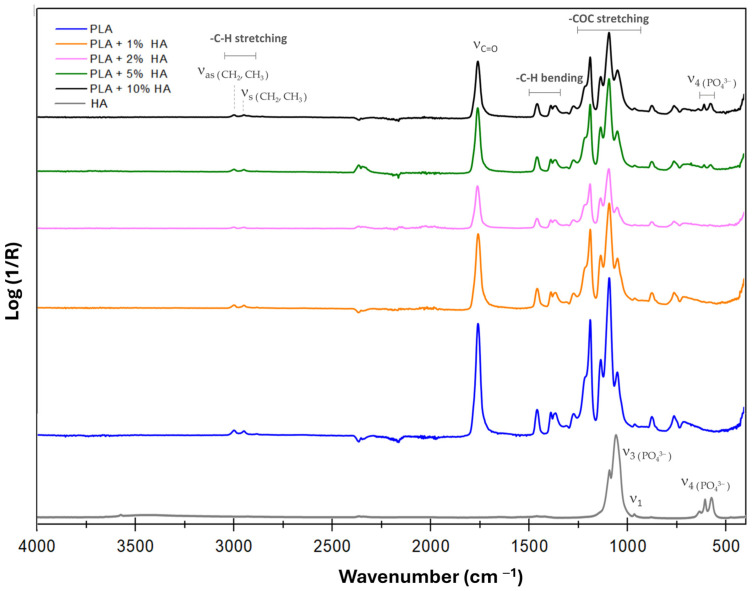
ATR-FTIR spectra of the PLA/HA samples.

In Figure 2, the ATR-FTIR spectra of the PLA/HA samples are shown. The spectra showed the absorption bands characteristic of both polylactic acid and hydroxyapatite. Specifically, in PLA, several key vibrational bands were identified, providing important information about its composition and structural characteristics. The region between 3000–2850 cm^−1^ shows C-H stretching bands, indicative of stretching vibrations associated with CH and CH_3_ groups that are present in the polymer’s backbone. A strong absorption band is observed in the vicinity of 1750–1755 cm^−1^, corresponding to the carbonyl group (C=O). The bands within the range of 1450–1380 cm^−1^ correspond to C-H bending vibrations, whereas ester groups reveal themselves through C-O stretching vibrations, detectable at 1180–1280 cm^−1^. The spectra also encompass bands in 1300–1000 cm^−1^, reflecting stretching vibrations linked to the C-C and C-O-C bonds within the polymer chain [42,43]. The main characteristic bands from HA were also present in the spectra [44,45,46]. According to the literature [45], in the 1200–500 cm^−1^ range, characteristic PO_4_^3−^ vibrations were expected, specifically, at ν_1_~960 cm^−1^ symmetric stretching mode of the PO_4_ tetrahedron [45,46], a strong doublet between 1091–1039 cm^−1^ usually is present and assigned to the asymmetric stretching (ν_3_) of the PO_4_^3−^ [45] while the bands at 570 cm^−1^ and 601 cm^−1^ corresponds to asymmetric bending modes [45] were detectable for the samples with higher particle content, given that the bands between 1000–1200 cm^−1^ overlapped with those from stretching vibrations of C-O-C bonds of the ester group and the rocking bands of the alkyl groups. 

Based on previous research work [37], the absorbance of the bands centered at 1055 cm^−1^ (ν_3_ (PO_4_)^3−^) should increase proportionally with the concentration of the nanofiller. However, only slight differences in the band ratio between the peaks at 1128 and 1041 cm^−1^, corresponding to the stretching vibrations of the C-O ester group, are observed. These changes in band ratio are detectable even for the sample filled with 1% HA NPs, but various factors can influence this band ratio apart from particle concentration. These factors may include changes in the conformation of rotational isomers (cis, trans) associated with the presence of nanoparticles and/or specific interactions. In addition to changes in the band ratio of the ester groups, only bands corresponding to bending modes (ν_4_ (PO_4_)^3−^) at 570 cm^−1^ and 601 cm^−1^ were detectable for the samples with higher particle content, i.e., PLA + 5% HA and PLA + 10% HA.

**Figure 3:**
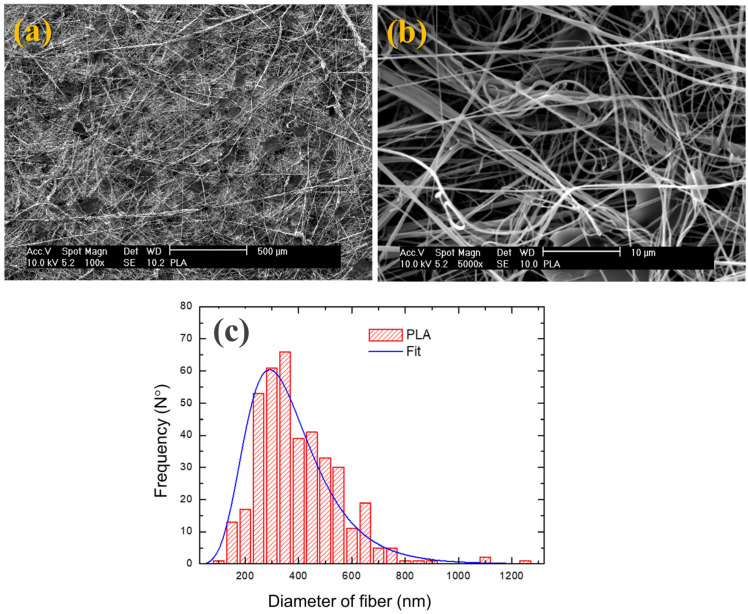
SEM images for pure polylactic acid (PLA) sample at (**a**) 100× and (**b**) 5000× magnifications. (**c**) Fiber diameter distribution for PLA.

Figure 3 presents the SEM images of pure PLA (low magnification 100×, Figure 3a and high magnification 5000×, Figure 3b. The images reveal a fibrillar morphology characterized by randomly distributed fibers. From the corresponding image analysis, the histogram of the distribution of fiber diameters (Figure 3c and the mean fiber diameter of 370 ± 140 nm were obtained.

**Figure 4:**
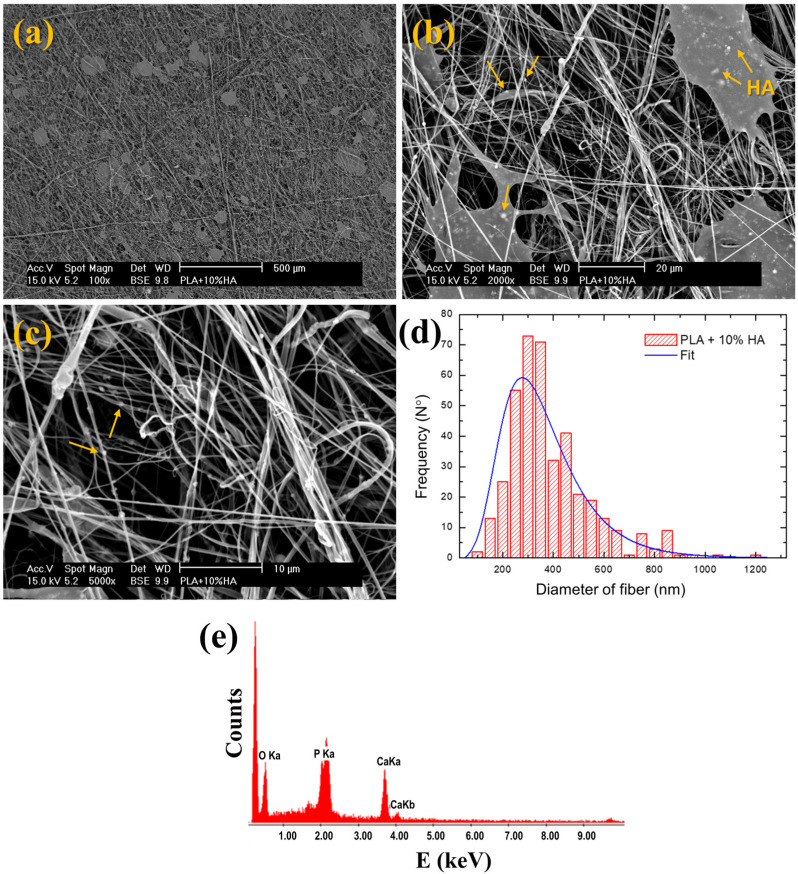
SEM images for polylactic acid (PLA) + 10% hydroxyapatite (HA) sample at (**a**) 100×, (**b**) 2000×, and (**c**) 5000×. (**d**) Fiber diameter distribution for PLA + 10% HA and (**e**) representative EDS microanalysis of hydroxyapatite-rich domains. (Note: The arrows point out HA rich domains).

A similar fibrillar morphology was observed for PLA filled with HA NPs. Figure 4 shows the SEM micrographs for PLA filled with 10% HA NPs. In particular, Figure 4a–c shows the morphologies observed at 100×, 2000× and 5000×, respectively. As shown in Figure 4a, platelet-like regions were observed distributed throughout the sample, which was attributed to a more abrupt ejection of the solution and the subsequent spreading of the sample due to air pressure. At 5000× (Figure 4c), only a characteristic region with fibrillar morphology of the PLA + 10% HA sample is shown. In Figure 4b,c, the arrows point out bright domains on the image, which are associated with the presence of HA particles, as was confirmed by energy-dispersive X-ray spectroscopy analysis. Given the particle content of this sample (10% HA NPs), in Figure 4b, there are bright spots showing domains of different sizes, indicating that for higher particle content, small aggregates of HA nanoparticles are formed. Nonetheless, the distribution of HA remains homogeneous throughout the sample. Figure 4d shows the distribution of the fiber diameters for the PLA + 10% HA sample, which exhibited a mean value of 357 ± 160 nm. Similar morphologies were observed for the rest of the samples under study (see Figure S1). In Figure 4e, a representative EDS microanalysis is shown, which corresponds to the HA-rich domains. The presence of calcium (Ca) and phosphorous (P) indicates the presence of HA. 

In Figure S1, the SEM images for the PLA/HA samples filled with 1%, 2%, and 5% NPs at different magnifications (100× and 5000×) are presented. More specifically, the morphologies for the samples PLA + 1% HA, PLA + 2% HA, and PLA + 5% HA are shown in Figure S1a and b, S1c and d, and S1e and f, respectively. As can be seen, regardless of the concentration of HA nanoparticles, the morphologies are very similar, showing mainly submicrometric fibers randomly oriented in all the samples. The low-magnification images showed a typical morphology of a non-woven mat where some platelet domains of sizes of several tens of microns are observed (see arrow in Figure S1a). On the other hand, the high-magnification images allow better visualization of the non-woven fibers obtained in all the blow-spun PLA/HA composites. To evaluate the effect of particle content in fiber morphology, image analysis was done, and the distributions of the diameter sizes of fibers were obtained. 

**Figure 5:**
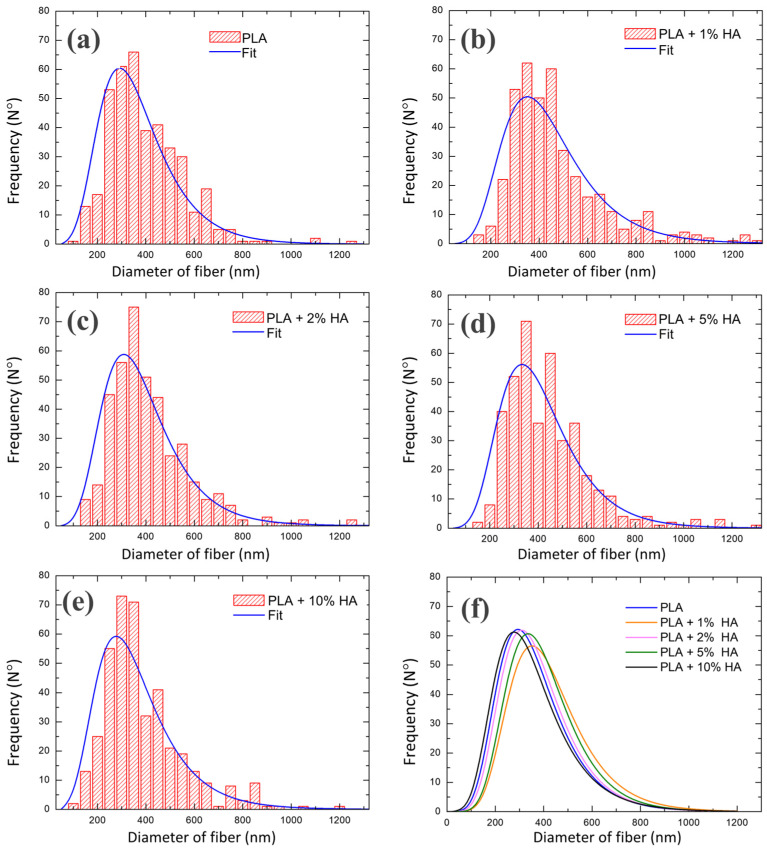
Fiber diameter and fiber diameter distributions of observed fiber diameters for (**a**) polylactic acid (PLA), (**b**) PLA + 1% HA, (**c**) PLA + 2% HA, (**d**) PLA + 5% HA, and (**e**) PLA + 10% HA and (**f**) fiber diameter distributions for the different polylactic acid (PLA) and PLA/hydroxyapatite (HA) nanocomposites.

In Figure 5, the histograms of the fiber diameters measured from the image analysis of the SEM images obtained at 5000× are shown in red. The mean values of the diameters for each material studied were also determined and included in Table 2. Table 2 lists the arithmetic average diameters of the fibers and the estimated porosities of the samples. The porosities were estimated from the specimens prepared to carry out the mechanical tests, as explained in Section 2.3. Regardless of the concentration of HA nanoparticles, the porosities of the samples were almost uniform at approximately 80%, except for that of pure PLA, which had a value of approximately 84%. Considering the differences found in the size of the fibers (Table 2), it can be inferred that, when adding HA nanoparticles, more dense non-woven mats are obtained. 

As can be seen in Table 2, the diameters of the fibers exhibited minimal variation with the composition. For example, slightly thinner fibers were obtained for pure PLA and PLA + 10% HA, with fiber diameters of less than 400 nm (370 ± 140 nm for PLA and 360 ± 160 nm for PLA + 10% HA), whereas slightly thicker fibers were obtained for the sample filled with 1% HA NPs (PLA + 1% HA), with an average diameter of 430 ± 170 nm. In this sample, a broader dispersion was also observed. The sample filled with 5% HA (PLA + 5% HA) exhibited an average fiber diameter of 400 ± 150 nm. In fact, slight variations in fiber diameter distributions were observed. For comparison, in Figure 5f, the estimated plots arising from the corresponding fittings of the fiber diameter distributions of all the samples are included. As can be observed, most samples have a similar diameter of fibers. Only slightly wider fibers were obtained for the sample filled with 1% HA. 

### 3.2. Thermal Characterization

**Figure 6:**
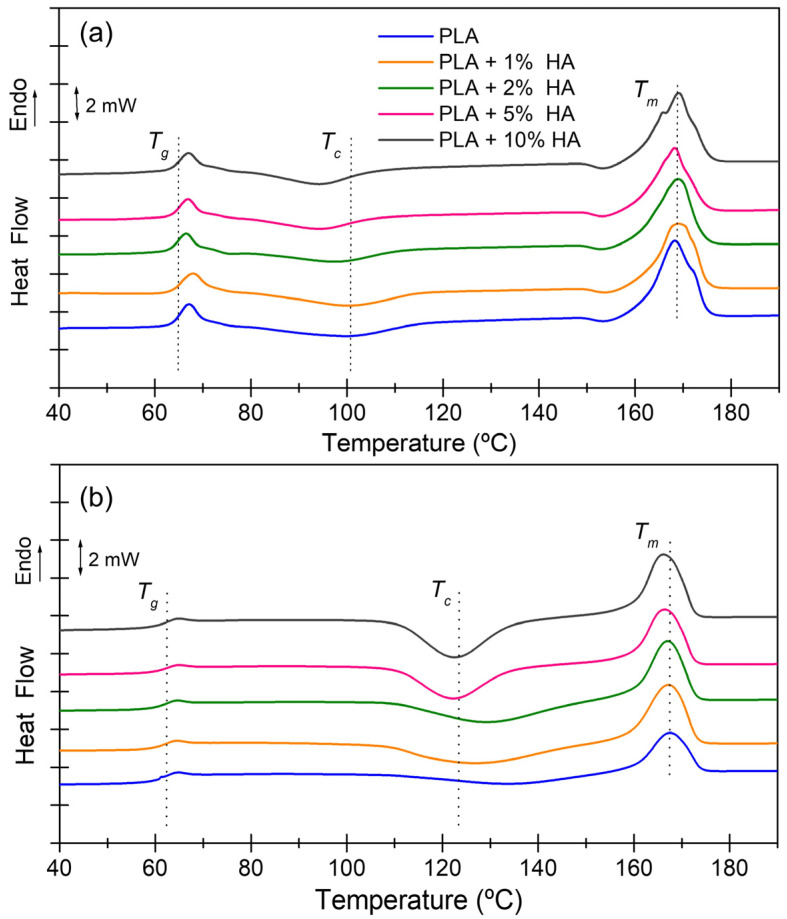
Differential scanning calorimetry thermograms of polylactic acid (PLA) and PLA/hydroxyapatite (HA) nanocomposites during (**a**) first and (**b**) second heating scan.

The influence of HA NPs addition on the thermal properties of PLA was studied using DSC. Figure 6 and Table 3 present the results of the first heating scan for the systems under investigation. 

The main transitions associated with the typical thermal behavior of PLA were observed [42,47,48,49]. Pure PLA displayed a glass transition temperature, *T*_g_, of 65.4 °C, an exothermic peak of cold crystallization, *T*_c_, at 100.5 °C, and an endothermic melting peak, *T*_m_, at 168 °C. Regardless of the HA content, *T*_g_ was similar in all samples: approximately 65.5 °C. The peak of the melting point also showed insignificant variation in the particle content. However, the cold-crystallization temperature and the corresponding energy released were clearly reduced with the addition of HA (Table 3). In the case of the enthalpy of this exothermic process, the clear decrease occurs until a concentration of HA of 2% since, from that point, less difference with respect to the neat PLA is observed the higher the concentration of HA nanoparticles (Table 3).

Increasing the particle content may favor the crystallization because of a nucleation effect, causing the crystallization process to occur at lower temperatures [50]. On the other hand, the energy released due to the cold crystallization process mainly depends on the ability of the material to yield new crystals, which, in turn, should depend on the crystalline degree of the material. The higher the initial fraction of crystals, the less ability for cold crystallization because less relative amount of amorphous phase is available to be crystallized. Therefore, two factors should be considered to explain the ability of the materials to generate crystals during cold crystallization: (i) nucleation and (ii) previous crystallinity, being the cold crystallization favored the higher the nucleation effect and the lower the initial crystallinity.. To evaluate these factors separately, the crystalline fraction before cold crystallization was estimated with Equation (1). As can be seen, except for the sample with 1% of HA, the crystallinity before cold crystallization is higher when adding HA nanoparticles; therefore, the most plausible explanation for the general decrease in the enthalpy for the cold crystallization must be the existence of less amorphous phase available for cold crystallization. 

On the other hand, although the melting temperature does not seem to be affected by the presence of the NPs (~168–169 °C), the shape of the endotherms slightly changes. As can be seen, a shoulder appears at lower temperatures (~165 °C) when the HA is added, which is enhanced the higher the concentration of HA is. However, this shoulder cannot be observed during the second heating scan (Figure 6b), suggesting a combination of factors to explain the appearance of that endothermic shoulder, the presence of nanoparticles, and the processing method, in this case, SBS. One possible explanation is that when fibers are formed during the SBS process, the presence of nanoparticles might force specific conformations of the polymer chains, and therefore, variations on the crystals are generated due to the presence of the nanoparticles [37,51]. 

Figure 6b and Table 4 present the results of the second heating scan for the systems under investigation. The main transitions associated with the typical thermal behavior of pure PLA were detected. However, some differences were observed with respect to the first heating scan, which were attributed to processing history. For example, the enthalpic relaxation at the glass transition (Figure 6a) was not observed in the second heating scan (Figure 6b). The presence of the enthalpic relaxation along the first heating scan must be due to residual stresses generated by the preferential direction associated with the polymer drawing when fibers are formed during the SBS process. However, during the second heating scan, after erasing the corresponding processing history, a more relaxed structure is evident. Specifically, the values of glass transition temperatures (*T_g_*) are lower for these more relaxed structures, as expected. Furthermore, the cold-crystallization temperature was higher during the second heating scan. However, the presence of the NPs reduced the cold-crystallization temperature during both the first and second heating scans, thus confirming its nucleating effect, as reported in previous works [50]. These results highlight a very interesting conclusion with significant implications for potential applications of fibrillar materials (scaffolds, filters, membranes, among others). The crystallinity fraction of the PLA-based materials produced by SBS, compared to those produced by other methods such as additive manufacturing [52], extrusion, or injection molding, can be increased even at lower temperatures, thereby reducing energy consumption while maintaining their fibrillar morphology.

### 3.3. Mechanical Characterization

The stress–strain curves for each of the specimens of each sample are gathered in Figure S2 (in the supplementary materials section). The curves show a similar behavior in each case. The variations observed between specimens of each sample can be explained by the complex microstructure of the materials. They are constituted by fibers, planar domains, and corpuscles or beads. Minor variations in the proportion of those microconstituents, their distribution, and, therefore, porosity might cause those differences observed in the profiles of the plots. However, the general shape and order of magnitude of the tensile stress–strain curves were like others reported in the literature for similar fibrous materials, especially electrospun materials [53].

**Figure 7:**
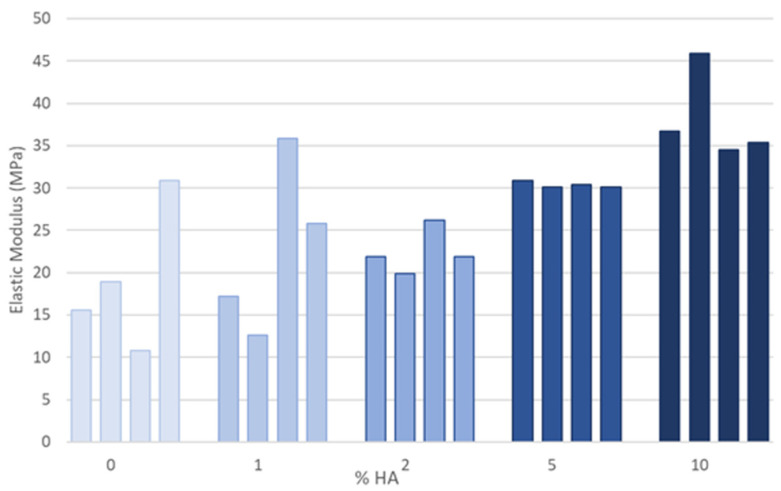
Representation of elastic modulus obtained for each specimen of polylactic acid/hydroxyapatite (HA) samples as a function of HA particle content.

Figure 7 shows the elastic modulus of each specimen tested in this study. The elastic modulus was obtained from the first linear part of the stress–strain plots. The orders of magnitude of the elastic moduli were like those observed in electrospun fibrous PLA materials. For example, Tjong et al. reported an elastic modulus of approximately 8.5 MPa for electrospun PLA and 10 MPa for electrospun PLA loaded with 15% nano-HA [50]. In this study, the elastic modulus for PLA + 10% HA was found to be 38.1 ± 5.3 MPa.

Values of Young’s modulus of all the materials are gathered in Table 5. The differences observed in the elastic moduli may be attributed to three factors: (i) concentration of HA particles, (ii) crystallinity, and (iii) morphology (size of fibers, proportion of microconstituents, and preferential orientation of microconstituents). As the concentration of HA particles increases, there should be an increase in Young’s modulus because PLA is being modified with a stiffer material. On the other hand, crystalline domains of PLA are also more rigid than amorphous regions; therefore, a higher proportion of crystalline regions should increase the elastic modulus. Finally, in terms of morphology, it is expected to have higher modulus and even mechanical strength the higher the proportion of fibers, mainly when they are aligned with the direction of load application. Mechanical tests can provide insight into the homogeneity of particle dispersion. Uneven dispersion of nano-reinforcement may negatively impact mechanical properties, including the elastic modulus and mechanical strength. Notably, incorporating 1% HA NPs resulted in improved elastic modulus and mechanical strength, indicating a uniform distribution within the matrix. Referred to the rest of the PLA/HA samples, if the dispersion were non-uniform or if particle aggregates or agglomerates existed, either no change or a reduction in mechanical properties of all the samples compared to those of the pure polymer would be expected.

The DSC experiments revealed that differences between the materials in terms of crystallinity do not follow any trend, while the elastic modulus increases as the concentration of particles increases. On the other hand, the morphology does not seem to change with the composition of materials, except in the case of neat PLA, for which the porosity is slightly higher, though it falls within standard deviation. Therefore, the differences in the elastic moduli were mainly attributed to the reinforcement effect caused by the presence of HA NPs. In previous studies focused on other nanocomposite materials, it was observed that for 2% of nanoparticles, there was a decrease in E with respect to the polymer-filled 1% NPs, probably because of the formation of aggregates in the composite material [37]. However, in the present study, this decrease in E was not observed, possibly because it was mitigated by an increase in the crystallinity from 24% to 31% (Table 3). 

Notably, the values of the elastic moduli were comparable with that of a spongy bone. Sydlyk et al. reported a modulus of 75 MPa for a spongy bone tested in compression mode [54]. Therefore, by modifying the HA loading and polymer crystallinity, which is also related to particle content, a similar modulus may be obtained for bone-related applications or other bio-related applications such as wound healing.

As can be seen, Table 5 also lists, apart from Young’s modulus, other characteristic parameters obtained from the mechanical tests: tensile strength, maximum strain at break, and total area under the curve. In addition, Figure S3 shows different plots where the values of parameters as a function of particle content are represented to aid the visualization and interpretation of the results. 

The elastic modulus increased with the particle content, as previously explained. The maximum strength was unaffected by the presence of NPs, at least for the materials under investigation. Strain and area under the curve parameters usually associated with the toughness of the materials show their maximum values when PLA is loaded with 1% of HA nanoparticles and then, they slightly decrease as the nanoparticle content increases. These results are consistent with the fact that the introduction of nanoparticles at concentrations higher than 1% highly increases the probability of the formation of aggregates and agglomerates that can favor stress accumulation at the interfaces, facilitating crack formation and, consequently, fragility.

Another possible explanation could be related to the morphologies of the samples. According to the mean values of diameters obtained in the morphological studies, slightly wider fibers were observed in PLA + 1% HA (430 ± 170 nm). This indicates that thick fibers favor ductility, thus leading to higher values of maximum strain (0.25 or 25% for the 1% HA sample). The PLA + 5% HA system contained thicker fibers, with diameters of approximately 400 ± 150 nm; however, the maximum strain was significantly lower than that for the sample filled with 1% HA. In the case of the PLA + 5% HA sample, the higher particle content may have increased the rigidity of the material, counteracting the potential ductility contributed by the diameter of the fibers. Furthermore, the thinnest fibers, those in PLA + 10% HA (360 ± 160 nm), exhibited a more brittle behavior, which could be ascribed to the effect of the particle content. This suggests a balance between morphology and particle content, which explains the observed maximum strain and area under the curve, the sample with 1% HA, the one that exhibits the maximum strain at break. In addition, in a previous work, it was proposed that, in polymer-reinforced materials, when there are enough nanoparticles, they can break apart from their aggregates and move freely through the polymer chains. This results in increased interactions between the particles, which ultimately allows for a greater amount of strain before failure [37].

The corresponding calculations using Equation (1) allow concluding that the degree of crystallinity before cold crystallization was null when the SBS processing history is erased by cooling from the melt at 10 °C/min. After cold crystallization, χ_c_ increased considerably for the samples filled with HA, varying between 22% for pure PLA and 38% for PLA + 10% HA. This indicated that the presence of NPs significantly affected the cold crystallization process if the initial PLA-based material was almost totally amorphous. However, this effect was not observed during the first heating scan. In fact, the variations in the degree of crystallinity of the samples during the first heating scan were insignificant, although the tendency was similar. The PLA + 10% HA sample exhibited the highest degree of crystallinity, probably due to the presence of the nanoparticles. 

## 4. Conclusions

In this study, non-woven PLA/HA materials were prepared via solution blow spinning (SBS), and their morphological, thermal, and mechanical properties were studied. The effect of particle content on the final properties of the materials was analyzed. Structural characterization by ATR-FTIR spectroscopy evidenced the presence of the HA nanoparticles. With increased particle content, the crystallinity of the materials increased slightly. Further, thermal characterization of the materials proved that the presence of HA NPs favored the crystallization of the polymer during thermal treatment. Mechanical characterization indicated that the presence of NPs increased the stiffness of the materials, and thereby increased their elastic moduli as expected, although their values were also affected by the crystallinities of the polymers. The morphologies of the fibers also affected the mechanical properties of the materials. Materials with larger fiber diameters exhibited maximum values of deformation at the break, suggesting more ductile behaviors. Also, the nanoparticles can interact with the polymer chains and favor the movement of the macromolecules, leading to larger deformations. Thus, the results of this study suggest a balance between the various factors that affect the mechanical properties of non-woven PLA/HA materials, including the fiber diameters of the SBS materials, degree of crystallinity, and particle content. The addition of HA NPs to PLA revealed that the presence of HA NPs affected (i) fiber diameter—thinner fibers were observed in pure PLA compared to PLA/HA nanocomposites; (ii) thermal behavior—increasing crystallinity with particle content; and (iii) mechanical properties—increasing elastic modulus and modifying deformation at break. However, given the complex microstructure of the materials, which included fibers, planar domains, and corpuscles or beads, in future studies, it is proposed to implement enhanced control measures during sample preparation and testing procedures to minimize the impact of microstructural variations. This will include refining processing parameters (for example, using different solvents or polymer concentrations) to ensure better morphological control, if possible, in terms of morphological microstructures. These factors should be controlled while preparing materials for potential applications in tissue engineering. 

In conclusion, our study has successfully demonstrated the preparation of polylactic acid fibers and hydroxyapatite composites using solution blow-spinning, offering valuable insights into the intricate relationship between material composition and mechanical properties. The observed changes in crystallinity and stiffness with increasing hydroxyapatite content, coupled with the influence of varying PLA fiber diameters on composite ductility, underscored the importance of carefully tuning these parameters. These findings not only contribute to a deeper understanding of composite behaviors but also suggest promising opportunities for advancing tissue engineering through the strategic manipulation of polylactic acid fibers, hydroxyapatite content, and crystallinity. These factors should be controlled while preparing materials for potential applications in tissue engineering.

## Figures and Tables

**Table 1:** Processing conditions for producing materials using SBS.

Pressure (bars)	Working Distance (cm)	Injection Rate (mL/min)	Collector Rotation Speed (rpm)	Volume Injected (mL)
2	11	0.25	200	25

**Table 2:** Average diameters and estimated porosities of the samples.

Sample	Diameter of Fiber (nm)	Estimated Porosity (%)
PLA	370 ± 140	84 ± 1
PLA + 1% HA	430 ± 170	80 ± 4
PLA + 2% HA	380 ± 150	80 ± 1
PLA + 5% HA	400 ± 150	80 ± 2
PLA + 10% HA	360 ± 160	80 ± 1

Note: Porosity values were estimated by comparing the density of each mixture with that of its corresponding bulk mixture.

**Table 3:** Differential scanning calorimetry data for first heating scan.

Sample	NPs (%)	*T*_g _ (°C)	*T*_c _ (°C)	*T*_m_ (°C)	Δ*H*_cc _ (J·g^−1^)	Δ*H*_m _ (J·g^−1^)	χ_c_ before ^1^ (%)	χ_c_ after ^2^ (%)
PLA	0	65.4	100.5	168.3	17.0	41.1	25.8	44.0
PLA + 1% HA	1	65.6	100.5	169.1	16.4	38.9	24.0	41.5
PLA + 2% HA	2	67.7	97.9	168.9	12.5	41.7	31.3	44.6
PLA + 5% HA	5	65.2	94.5	168.2	14.6	39.0	26.1	41.7
PLA + 10% HA	10	65.2	94.4	169.1	16.5	43.3	28.6	46.3

Note: PLA, polylactic acid; HA, hydroxyapatite; NP, nanoparticle; *T*_g_, glass transition temperature; *T*_c_, cold-crystallization temperature; *T*_m_, melting temperature; Δ*H*_cc_, cold-crystallization enthalpy; Δ*H*_m_, melting enthalpy; χ_c_, degree of crystallinity; ^1^ Degree of crystallinity as defined in Equation (1); ^2^ Degree of crystallinity considering all crystals (Δ*H*_m_/Δ*H*_reference_).

**Table 4:** Differential scanning calorimetry data for second heating scan.

**Sample**	**NPs** **(%)**	***T*** **_g_** **(°C)**	***T*** **_c_** **(°C)**	***T*** **_m_** **(°C)**	Δ***H*_cc_** **(J·g^−1^)**	Δ***H*_m_** **(J·g^−1^)**	**χ_c_** ** after ^2^** **(%)**
PLA	0	60.7	134.2	167.5	20.2	20.1	21.5
PLA + 1% HA	1	62.2	127.3	167.2	40.8	30.8	32.9
PLA + 2% HA	2	62.4	129.5	167.1	42.8	31.3	33.4
PLA + 5% HA	5	62.5	122.3	166.5	45.9	29.0	30.9
PLA + 10% HA	10	62.6	122.7	166.3	43.6	35.9	38.3

Note: PLA, polylactic acid; HA, hydroxyapatite; NP, nanoparticle; *T*_g_, glass transition temperature; *T*_c_, cold-crystallization temperature; *T*_m_, melting temperature; Δ*H*_cc_, cold-crystallization enthalpy; Δ*H*_m_, melting enthalpy; χ_c_, Degree of crystallinity as defined in Equation (1); ^2^ Degree of crystallinity considering all crystals (Δ*H*_m_/Δ*H*_reference_).

**Table 5:** Average mechanical properties obtained from the uniaxial tension tests.

Sample	NPs (%)	Elastic Modulus (MPa)	Tensile Strength (MPa)	Maximum Strain (1/1)	Area under Curve (MPa)
PLA	0	19.0 ± 9.0	1.07 ± 0.43	0.15 ± 0.03	0.093 ± 0.05
PLA + 1% HA	1	23.0 ± 10	1.28 ± 0.31	0.25 ± 0.08	0.153 ± 0.05
PLA + 2% HA	2	22.5 ± 3.0	1.01 ± 0.35	0.14 ± 0.04	0.082 ± 0.05
PLA + 5% HA	5	30.4 ± 0.4	1.20 ± 0.37	0.13 ± 0.04	0.105 ± 0.06
PLA + 10% HA	10	38.0 ± 5.5	1.07 ± 0.44	0.12 ± 0.08	0.099 ± 0.09

## Data Availability

Data are available upon request.

## Supplementary Materials

The following supporting information can be downloaded at https://www.mdpi.com/article/10.3390/nano14020196/s1, Figure S1: SEM images of various polylactic acid/hydroxyapatite (PLA/HA) samples: (a,b) PLA + 1% HA; (c,d) PLA + 2% HA; and (e,f) PLA + 5% HA at 100× (left) and at 5000× (right); Figure S2: Stress–strain curves for polylactic acid (PLA) and PLA/hydroxyapatite (HA) nanocomposites: (a) PLA, (b) PLA + 1% HA, (c) PLA + 2% HA, (d) PLA + 5% HA, and © PLA + 10% HA; Figure S3: Average mechanical properties obtained for polylactic acid/hydroxyapatite (HA) systems as functions of HA particle content: (a) Elastic Modulus, E (MPa); (b) Tensile strength (MPa); (c) Maximum strain (1/1); and (d) Area under the curve (MPa).
