# Supplementary material for: Non-Woven Fibrous Polylactic Acid/Hydroxyapatite Nanocomposites Obtained via Solution Blow Spinning: Morphology, Thermal and Mechanical Behavior
Source: Nanomaterials (Basel). 2024 Jan 15;14(2):196. doi: 10.3390/nano14020196 (PMC10818388; doi:10.3390/nano14020196)
Supplement: Supplementary file 1 [file nanomaterials-14-00196-s001.zip › nanomaterials-2819353-supplementary.pdf]

nanomaterials

Supplementary Materials

Non-Woven Fibrous Polylactic Acid/Hydroxyapatite Nanocomposites Obtained via Solution Blow Spinning: Morphology, Thermal and Mechanical Behavior

Javier González-Benito [1,2], Stephania Zuñiga-Prado [1], Julian Najera [3] and Dania Olmos [1,2,*]

[1] Department of Materials Science and Engineering and Chemical Engineering, Instituto de Química y Materiales Álvaro Alonso Barba (IQMAA), Universidad Carlos III de Madrid, Avda. Universidad 30, 28911 Leganés, Madrid, Spain; javid@ing.uc3m.es (J.G.-B.); stzuniga@ing.uc3m.es (S.Z.-P.)
[2] Instituto de Química y Materiales Álvaro Alonso Barba (IQMAA), Universidad Carlos III de Madrid, Avda. Universidad 30, 28911 Leganés, Madrid, Spain;
[3] Department of Aerospace & Mechanical Engineering, University of Notre Dame, Notre Dame, IN 46556, USA; jnajera2@nd.edu
* Correspondence: dolmos@ing.uc3m.es

3.1. Structural and Morphological Characterization

Figure S1 shows the SEM images for the PLA/HA samples filled with 1%, 2% and 5 % NPs at different magnifications (100× and 5000×).

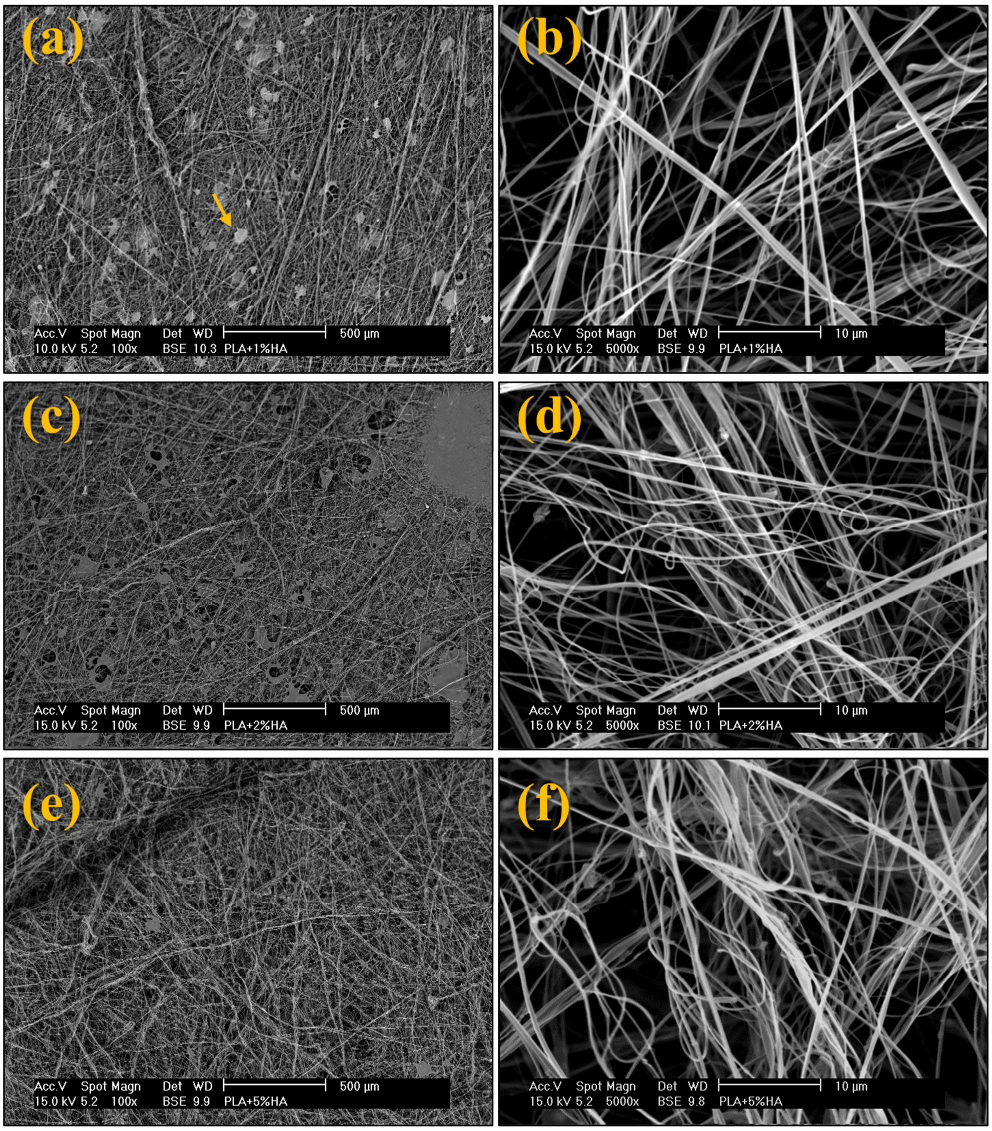

Figure S1. SEM images of various polylactic acid/hydroxyapatite (PLA/HA) samples: a), b) PLA + 1% HA; c), d) PLA + 2% HA and e), f) PLA + 5% HA at 100× (left) and at 5000× (right).

3.3. Mechanical Characterization

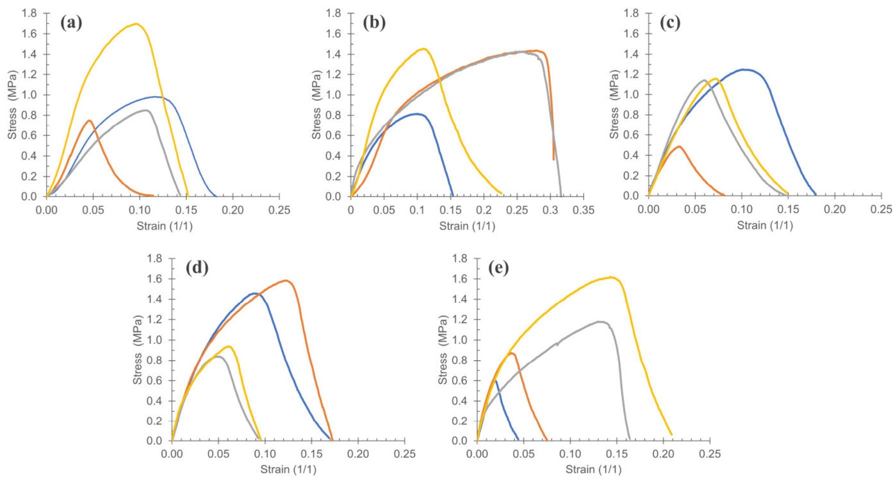

Figure S2. Stress–strain curves for polylactic acid (PLA) and PLA/hydroxyapatite (HA) nanocomposites: (a) PLA, (b) PLA+1% HA, (c) PLA+2% HA, (d) PLA+5% HA, and (e) PLA+10% HA.

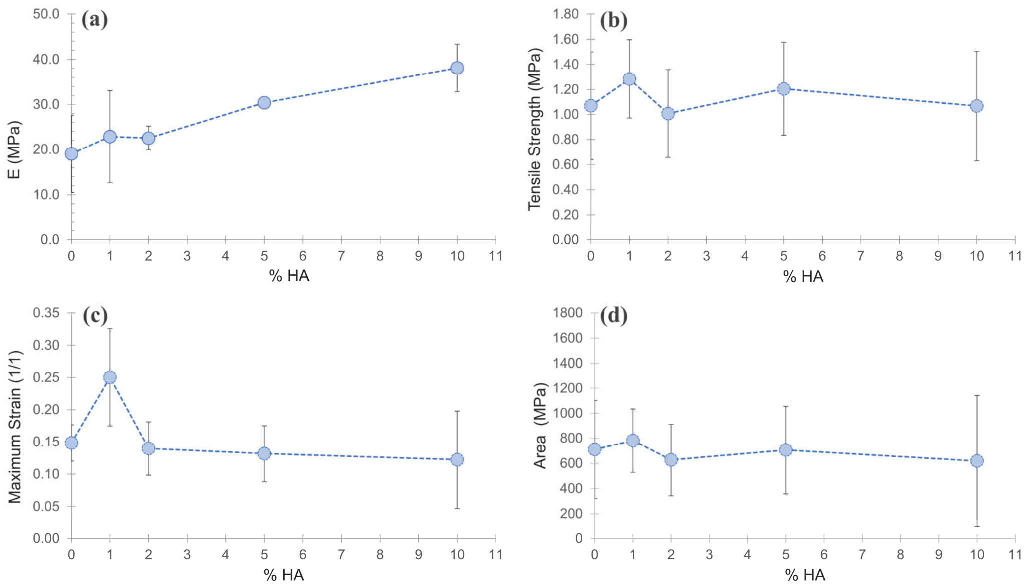

Figure S3. Average mechanical properties obtained for polylactic acid/hydroxyapatite (HA) systems as functions of HA particle content: a) Elastic Modulus, E (MPa); b) Tensile strength (MPa); c) Maximum strain (1/1) and d) Area under the curve (MPa).
